# Automated tools for evidence quality assessment: a scoping review

**DOI:** 10.1186/s12874-026-02868-3

**Published:** 2026-06-15

**Authors:** Jiayi Huang, Xinxin Deng, Liying Zhou, Junliang Tao, Cui Liang, Kehu Yang, Xiuxia Li

**Affiliations:** 1 Health Technology Assessment Center/Evidence-Based Social Science Research Center, School of Public Health, Lanzhou University, 199 Donggang West Road, Lanzhou, 730000 China; 2 Industrial Technology Center for Medical Big Data and Health Technology Assessment of Gansu Province, Lanzhou, 730000 China; 3 Key Laboratory of Evidence Based Medicine and Knowledge Translation of Gansu Province, Lanzhou, 730000 China; 4 Institute of Medical Information, Chinese Academy of Medical Sciences and Peking Union Medical College, Beijing, 100020 China; 5 Center for Clinical Big Data and Statistics of the Second Affiliated Hospital Zhejiang University School of Medicine, School of Public Health Zhejiang University School of Medicine, Hangzhou, 310000 China

**Keywords:** Evidence Quality Assessment, Scoping Review, Critical Appraisal, GRADE, Automated Tools, Decision Support, Public Health Decision-Making

## Abstract

**Background:**

Evidence quality assessment is critical for informed public health decision-making, but manual approaches are time-consuming and subject to variability. Automated support tools have been proposed to improve efficiency and consistency, yet their current status has not been comprehensively or systematically mapped.

**Objective:**

To identify or map the characteristics, performance, and limitations of existing automated tools for evidence quality assessment.

**Methods:**

Following the JBI methodology and PRISMA-ScR checklist, we searched 6 English and 4 Chinese databases from their inception to February 9, 2025, to identify studies evaluating automated tools for evidence quality assessment. Eligible studies included original research on tool development, application, or validation. Study characteristics (e.g., year, country, design, tool type, technical features, reliability, and validity) were extracted and summarized descriptively.

**Results:**

Twenty studies were included, most from the United Kingdom (30%), Canada (25%), and Australia (15%). Observational designs predominated (75%), with only 10% randomized controlled trials (RCTs). Twelve distinct tools were identified, of which 65% were publicly available. 58% of the tools were developed for RCTs, while 50% remained experimental and required human oversight. Reported outcomes focused on sensitivity, specificity, precision, efficiency, and consistency. Despite promising results, external validity and scalability were limited.

**Conclusion:**

Automated tools for evidence quality assessment show potential to enhance efficiency and consistency but remain restricted in applicability. Current tools are often tailored to clinical trials and require human supervision. Broader adaptation and rigorous validation are needed before such tools can be widely integrated into public health decision-making.

**Supplementary Information:**

The online version contains supplementary material available at 10.1186/s12874-026-02868-3.

## Introduction

Evidence-based practice in public health has received attention in recent years, particularly in the post-pandemic era. According to our systematic search, as of January 2025, 25,949 publications indexed in Web of Science included both “public health” and “systematic review” as topics, with 60% published in the past five years. Evidence-based decision-making has made high-quality systematic reviews a cornerstone of public health practice, with decision-makers increasingly reliant on robust evidence [1, 2]. However, the utility of these systematic reviews depends on the underlying evidence quality. Evidence quality assessment refers to the systematic assessment and classification of research studies based on methodological rigor, risk of bias, and the overall certainty of their findings [3–5]. Such assessment is essential for distinguishing trustworthy findings from misleading ones and guiding resource allocation. Nevertheless, the expanding body of literature poses major challenges for timely evidence selection and appraisal to inform decision-making.

Traditional manual review processes are labor-intensive and time-consuming. The average duration to complete a systematic review is approximately 11 months, and assessing risk of bias requires nearly 20 min per study, often involving multiple reviewers [6, 7]. Such processes reduce timeliness and are further limited by variability introduced through subjective judgments [8, 9].

Artificial intelligence (AI) has been increasingly applied to streamline evidence synthesis. Natural language processing (NLP) and machine learning (ML) methods have been used in study classification, screening, data extraction, and evidence appraisal [10–12]. Automated approaches for risk-of-bias assessment are particularly advanced. These methods extract data elements relevant to appraisal standards and classify studies accordingly [13]. Semi-automated tools that combine machine recommendations with human verification have demonstrated substantial gains in efficiency and consistency [14, 15]. In the medical and clinical research domains, several automated tools for evidence quality assessment have been developed and implemented. For instance, STEED was primarily developed for data mining in neuroscience in vivo publications and performs well in parameter extraction [16], and RobotReviewer employs machine learning algorithms to automatically assess risk of bias (ROB) in randomized controlled trials (RCTs) using the Cochrane ROB tool [17]. Nevertheless, challenges remain in generalizability and reliability across disciplines, and no automated tool for evidence quality assessment has been specifically tailored to the public health domain. Existing scoping reviews have mapped general automated tools for systematic review processes, but they did not specifically address evidence quality assessment [18, 19]. The present study focuses exclusively on automated tools for evidence quality assessment to inform the development of automated tools for evidence quality assessment in public health decision-making.

This study aimed to identify automated tools for evidence quality assessment. Four dimensions—effectiveness, reliability, user feedback, reported limitation—were chosen. These dimensions are critical for presenting the accuracy and reliability of automated tools for evidence quality assessment, and they reflect the key outcomes typically reported in studies on evidence appraisal tools. So, the objectives were to (1) summarize the technical approaches and applications of current tools, (2) identify or map core indicators such as adaptability, reliability, and validity reported in the included studies, and (3) identify limitations of existing automated tools themselves.

## Methods

This scoping review was conducted in accordance with JBI methodology for scoping reviews [20], and reported based on the Preferred Reporting Items for Systematic Reviews and Meta-Analyses extension for Scoping Reviews (PRISMA-ScR) Checklist [21], and the checklist is provided in Additional file 1.

### Registration

The scoping review protocol was registered in Open Science Framework (retrieved from osf.io/gfhsa). 

### Eligibility criteria

Studies were eligible if they reported on automated tools for evidence quality assessment. Automated tools were defined as computer-based technologies that utilize artificial intelligence, machine learning, natural language processing, or rule-based algorithms to perform tasks traditionally requiring human judgment, with minimal or no human intervention in the assessment process. Evidence quality assessment was defined as systematic assessment and classification of research studies based on methodological rigor, risk of bias, and the overall certainty of their findings, including ROB, evidence quality assessment (EQA), reporting quality assessment (RQA), or methodological quality assessment (MQA). Both quantitative and qualitative designs (e.g., experimental, observational, descriptive, and evaluative studies) published in Simplified Chinese or English were included [22]. Grey literature was not searched, as this review focused on published articles in peer-reviewed journals that employ scientific methods and evidence-based conclusions. Exclusion criteria: (1) studies lacking automated tools (e.g., manual-only assessment) or unrelated to the topic of automated tools; (2) automated tools applied to non-evidence quality assessment (e.g., clinical diagnosis, drug discovery, outcome prediction, sleep quality assessment, or product quality assessment); (3) only algorithm studies: pure algorithm studies without corresponding tools for application or validation; (4) duplicate or retracted publications; (5) non–peer-reviewed materials such as conference abstracts, news articles, policy documents, editorials, or books; and studies with insufficient data.

### Data sources and search strategy

Six English databases (PubMed, Web of Science, Cochrane Library, Embase, IEEE Xplore, and ACM Digital Library) and four Chinese databases (Chinese Biomedical Literature Database, China National Knowledge Infrastructure, Wanfang Data, and VIP) were searched from inception through February 9, 2025. Search strategies combined subject headings and free-text terms. Chinese search terms included synonyms for evidence quality assessment and artificial intelligence. English search terms included (“GRADE approach” OR “evidence grading system” OR “GRADE assessment” OR “evidence level” OR “quality assessment”) AND (“algorithm*” OR “artificial intelligence” OR “machine learnin*” OR “deep learnin*” OR “large language model”). Reference lists of included studies were also hand searched. Full search strategies are provided in Additional file 2 Table S1.

### Study selection

Records were managed in EndNote 20 [23]. After automated removal of duplicates, 2 reviewers independently screened titles and abstracts, retrieved potentially eligible full texts, and assessed eligibility against inclusion criteria [24, 25]. Screening decisions were based on the eligibility criteria outlined above, specifically focusing on: (1) the presence of automated tools (AI, machine learning, natural language processing, or rule-based algorithms) with minimal human intervention; (2) application to evidence quality assessment tasks (ROB, EQA, RQA, or MQA); and (3) exclusion of pure algorithm studies without evidence quality assessment validation, non-peer-reviewed materials, and studies lacking sufficient data. Discrepancies were resolved through discussion or consultation with a third reviewer. Prior to formal screening, a pilot calibration exercise was conducted on a random sample of 50 records to ensure reviewer consistency and refine the application of eligibility criteria.

### Data extraction

Two reviewers independently extracted data using a standardized form adapted from Pollock et al. [26]. The extraction items included bibliographic information (author, year, country) and study characteristics (design, type). Specific adaptations included adding detailed items on tool characteristics (tool name, methodological standard, technology implemented, intended task, application context), performance metrics (e.g., reliability, validity) and reported limitations to capture the features of automated tools. Any disagreements were resolved by consensus or adjudication by a third reviewer.

### Data analysis

Descriptive statistics were performed using Microsoft Excel 2016 [27]. The purpose of this analysis was to characterize the basic features of included studies and tools. Categorical variables were defined as country, study type, tool name, and methodological standard, and were analyzed using frequencies (n) and percentages (%). To describe the technical and functional attributes of the tools, technology implemented, intended task, and application context were summarized narratively. In addition, a narrative synthesis was conducted to summarize evidence on tool performance, including effectiveness, reliability, user feedback and reported limitations four dimensions. (1) The effectiveness refers to the ability of automated tools to correctly assess the quality of evidence, which was evaluated based on key indicators such as sensitivity, specificity, positive and negative predictive values (PPV and NPV), as well as positive and negative likelihood ratios (LR + and LR−). (2) The reliability refers to the consistency and stability of automated tools in assessing the quality of evidence, including percent agreement, intraclass correlation coefficient (ICC), Cohen’s kappa coefficient, and Cronbach’s alpha coefficient, etc. (3) The user feedback refers to subjective evaluations from users regarding the usability, practicality, efficiency, and overall experience of automated evidence quality assessment tools. (4) The reported limitations refer to the limitations of existing automated tools themselves such as application scale, applicable language, applicable study design. Findings were synthesized to provide an overview of current evidence.

## Results

### Study selection

**Fig. 1:**
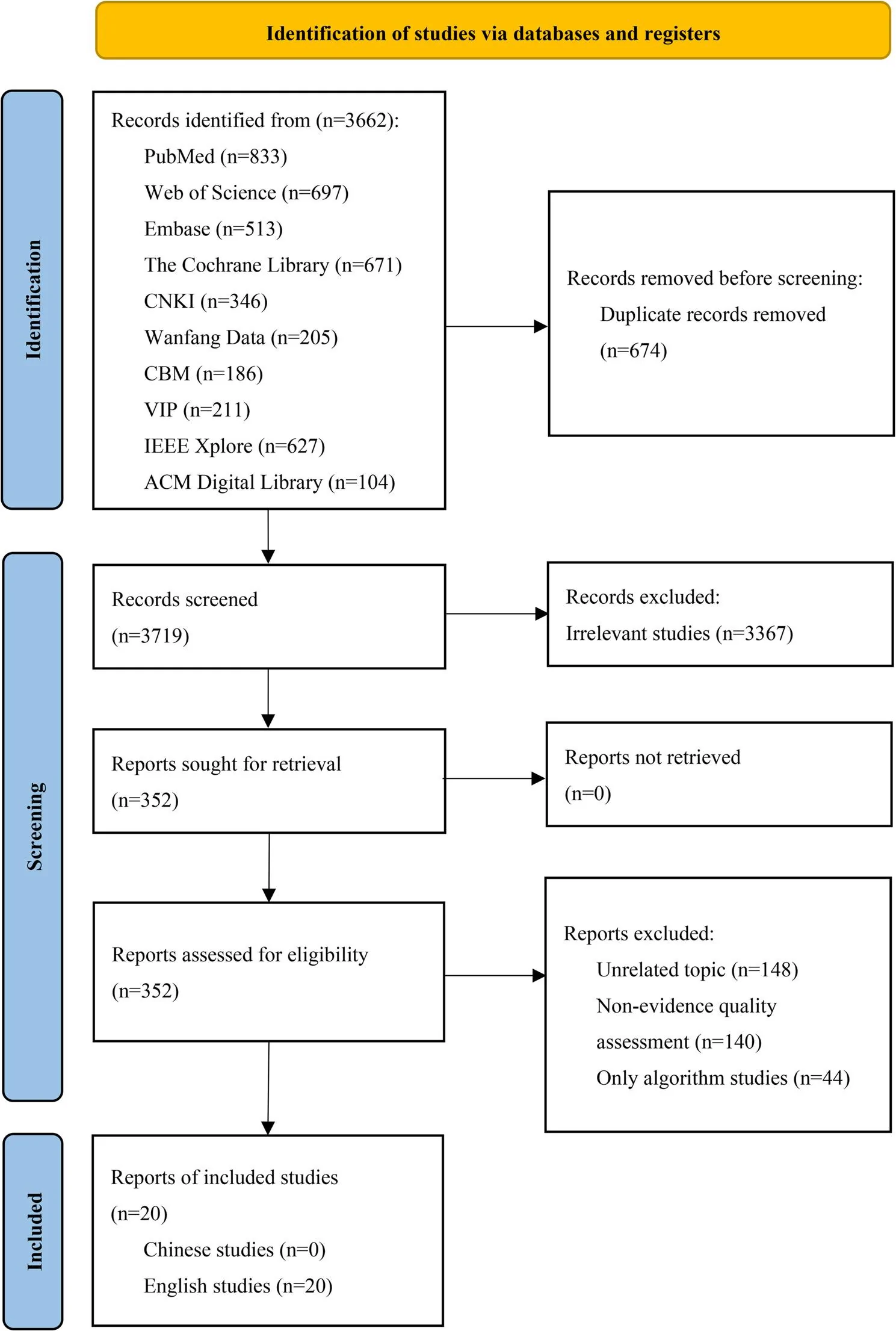
PRISMA 2020 flow diagram of the literature screening process and results [28]. CNKI: China National Knowledge Infrastructure; CBM: Chinese Biomedical Literature Database

Database searches identified 948 Chinese and 3445 English records. After deduplication, 3719 records were screened by title and abstract. At this stage, 3367 records were excluded as they clearly did not meet the predefined eligibility criteria. Full texts of the remaining 352 articles were reviewed for eligibility. Of these, 332 were excluded based on the following exclusion criteria: (1) being unrelated to the topic of automated tools (*n* = 148); (2) non-evidence quality assessment (*n* = 140), including studies focusing on image quality, fruit quality, product quality, and sleep quality; (3) only algorithm studies (*n* = 44), specifically studies presenting algorithms only without corresponding tools for application or validation. A total of 20 studies were included for analysis (Fig. 1).

### Study characteristics

Of the 20 included studies, 15 (75%) were observational, 3 (15%) were methodological, and 2 (10%) were randomized controlled trials. Research in this field first appeared in 2008, followed by a 5-year gap. From 2014 to 2017, approximately one study was published annually, and between 2020 and 2024, the number of publications increased to 2–3 studies per year. Most studies originated from countries with relatively early involvement in artificial intelligence research and development, including the United Kingdom (*n* = 6), Canada (*n* = 5), Australia (*n* = 3), and Sweden (*n* = 3). The basic characteristics of the included studies are shown in Additional file 2 Table S2.

### Types of automated support tools

Across 20 studies, 12 distinct tools for automated evidence quality assessment were identified; 65% were reported as publicly available, while 35% did not specify accessibility: (1) ROB assessment: RobotReviewer (*n* = 9) [17, 29–36], Evidence Mapping Tool (*n* = 1) [37], ExaCT (*n* = 1) [38], and STEED (*n* = 1) [16] primarily targeted ROB assessment; ROB 2 Excel Macro Tool (*n* = 1) [39], ChatGPT (*n* = 1) [40], and Claude 2 (*n* = 1) [41] primarily targeted ROB 2 assessment. These tools applied techniques such as machine learning algorithms (binary classification) and natural language processing to highlight relevant text fragments and semi-automated extraction of trial data, improving efficiency in bias detection. (2) EQA: EvidenceGRADEr (*n* = 1) [42], GRADEpro (*n* = 1) [43], and another not named tool (*n* = 1) [44] were developed for evidence quality assessment. These tools used classifiers to predict evidence grading levels and generated evidence summary tables to optimize the EQA process. (3) MQA, RQA: APPRAISE-AI (*n* = 1) [45] and large language model–based tools (*n* = 1) [46] integrated AI with expert judgment to evaluate methodological rigor and reporting quality. For detailed information on the technology implemented, intended task, and application context of each tool, see Additional file 2 Table S2.

### Performance evaluation

Tool performance was mapped using metrics such as specificity, sensitivity, accuracy, precision, consistency, reliability, recall, F1 scores, and time savings reported in the included studies. STEED and ExaCT demonstrated high effectiveness, whereas RobotReviewer, EvidenceGRADEr, and APPRAISE-AI were rated as moderate. STEED also showed high reliability, while 50% of the tools were rated as moderate. User feedback was reported as moderate for GRADEpro, ChatGPT, Automatic Evidence Quality Prediction, and RobotReviewer; no user feedback data were available for several other tools. Overall, 50% of the tools demonstrated potential for efficiency gains but remained semi-automated (Table 1).

**Table 1:** Summary of automated support tools for evidence quality assessment

Tool	Strengths			Reported limitations*
	Effectiveness	Reliability	User feedback	
Robot Reviewer	㊉㊉	㊉㊉	㊉㊉	①②④
EvidenceGRADEr	㊉㊉	㊉㊉	㊀	②③
Automatic evidence quality prediction	㊉㊉	㊀	㊉㊉	⑤
GRADEpro	㊀	㊉㊉	㊉㊉	①
APPRAISE-AI	㊉㊉	㊉㊉	㊀	⑤
ChatGPT	㊉㊉	㊉㊉	㊉㊉	①
STEED	㊉㊉㊉	㊉㊉㊉	㊀	⑤
Large language models	㊉㊉	㊀	㊀	①
ExaCT	㊉㊉㊉	㊀	㊀	⑤
Claude 2	㊉㊉	㊀	㊀	①
Evidence mapping tool	㊉㊉	㊀	㊀	②③
ROB 2 Excel Macro Tool	㊉㊉	㊉㊉	㊀	①②

㊉㊉㊉ High: strong positive results reported; ㊉㊉ Moderate: partial positive results; ㊉ Low: negative or limited results; ㊀ Not reported: no relevant data available

Reported limitations: ① Requires human supervision for semi-automated assessment; ② Applicable only to RCTs; ③ Limited to small-scale studies; ④ Applicable only to English-language publications; ⑤ Limitations not reported

### Limitations of existing tools

58% of the tools were evaluated only in randomized controlled trials, with limited applicability to observational, qualitative, or mixed-methods studies. The majority of studies focused on medical evidence, with few applications in education, social sciences, or public health. Reported comparisons between automated and manual assessments of time and accuracy were inconsistently quantified. 50% of the tools required human oversight and were not fully automated. In addition, 17% of the tools were developed for small-scale studies or narrow datasets, reducing their generalizability to broader and more complex research contexts (Table 1).

## Discussion

This review identified 12 automated support tools for evidence quality assessment, with 70% of the research originating from the United Kingdom, Canada, and Australia. These tools addressed risk-of-bias assessment, methodological quality appraisal, and reporting evaluation. However, many remain experimental and lack maturity or widespread implementation.

Artificial intelligence–based tools have the potential to streamline evidence appraisal by applying standardized frameworks. Among the tools reviewed, RobotReviewer was the most extensively studied. Using natural language processing, it automatically extracts and analyzes trial information to generate risk-of-bias assessments, with reported accuracy ranging from 71% to 78% [33]. While time-saving, its reliability remains uncertain; some studies reported lower sensitivity compared with human reviewers, raising concerns about its robustness [35]. Comparative evaluations across different tools are limited, and performance measures lack standardization. Although 25% of the automated tools for assessing risk of bias have demonstrated better performance in terms of consistency, their methodological standards are derived from ROB, and there is still no comparably innovative instrument has been developed specifically for the revised ROB 2. Other tools—including the Evidence Mapping Tool, ExaCT, STEED, ROB 2 Excel Macro Tool, EvidenceGRADEr, APPRAISE-AI, GRADEpro, and large language model–based approaches such as ChatGPT and Claude 2—demonstrate varying strengths. STEED and ExaCT showed favorable validity, whereas GRADEpro and ChatGPT-based applications received positive user feedback. Nevertheless, 50% of the tools remain semi-automated, requiring human supervision and intervention [17, 47]. Performance evaluations typically focus on specificity, precision, accuracy, efficiency, sensitivity, and reliability. Scholars have suggested that composite measures such as the F-score or the area under the receiver operating characteristic curve (AUC) may better capture overall performance than single metrics, and could provide a standardized basis for future comparisons [48–50].

Despite their potential to improve efficiency and save time, current tools face several limitations. First, 50% of the tools require human oversight and cannot yet operate fully autonomously. Second, no unified framework or standards exist, restricting comparability and widespread adoption. Third, many tools lack user-friendly interfaces, limiting accessibility for nontechnical researchers despite promising algorithmic performance [51–53]. Fourth, 33% of the tools are restricted to English-language publications and randomized clinical trials, reducing their generalizability. Future development should focus on advancing fully automated tools to reduce dependence on manual oversight, establishing standardized frameworks for evaluation, enhancing user-friendliness to broaden accessibility, and expanding applicability across languages and study types to support global public health decision-making.

This study has limitations. Findings were derived primarily from published literature, and no primary validation of tools was conducted, which may introduce bias. Due to the unclear comparability of user populations, cross-tool comparisons of user feedback results should be interpreted cautiously. In addition, only English- and Chinese-language publications were included, potentially excluding relevant evidence from other languages.

## Conclusion

This review identified 12 artificial intelligence–assisted tools for evidence quality assessment, including RobotReviewer, STEED, and EvidenceGRADEr, with functions spanning methodological and reporting quality appraisal. Although these tools show considerable potential to improve efficiency and reduce manual effort, most remain experimental and dependent on human oversight, and none have achieved full automation. Nevertheless, they demonstrate promising prospects for future application, particularly in enhancing the efficiency and streamlining of evidence quality assessment.

## Supplementary Information

Additional file 1.

Additional file 2.

## Data Availability

No datasets were generated or analysed during the current study.

## Abbreviations

RCTs: Randomized controlled trials
AI: Artificial intelligence
NLP: Natural language processing
ML: Machine learning
PRISMA: Preferred Reporting Items for Systematic Reviews and Meta-Analyses
PRISMA-ScR: Preferred Reporting Items for Systematic Reviews and Meta-Analyses extension for Scoping Reviews
ROB: Risk of bias
EQA: Evidence quality assessment
RQA: Reporting quality assessment
MQA: Methodological quality assessment
GRADE: Grading of Recommendations, Assessment, Development and Evaluations
