# Supplementary material for: Automated tools for evidence quality assessment: a scoping review
Source: BMC Med Res Methodol. 2026 Jun 15;26:187. doi: 10.1186/s12874-026-02868-3 (PMC13495222; doi:10.1186/s12874-026-02868-3)
Supplement: Supplementary file 2 [file 12874_2026_2868_MOESM2_ESM.docx]

**Supplementary Appendix**

TableS1 Search strategy for automated support tools for evidence quality assessment

| **PubMed database <Feb 9, 2025>** | | **Results** |
| --- | --- | --- |
| #1 | “artificial intelligence”[MeSH Terms] OR “machine learning” [MeSH Terms] OR “neural networks” [MeSH Terms] | 214,146 |
| #2 | “algorit*”[Title/Abstract] OR “artificial intelligence”[Title/Abstract] OR AI[Title/Abstract] OR “computational intelligence”[Title/Abstract] OR “machine intelligence”[Title/Abstract] OR “computer reasoning”[Title/Abstract] OR “computer vision*”[Title/Abstract] OR “machine learnin*”[Title/Abstract] OR “large language model” [Title/Abstract] OR “deep learnin*”[Title/Abstract] OR “neural network*”[Title/Abstract] OR “computer-assisted”[Title/Abstract] OR “computer-aided”[Title/Abstract] OR “support vector machine*”[Title/Abstract] OR “support vector network*”[Title/Abstract] OR “computer methodologies”[Title/Abstract] OR “automated pattern recognition”[Title/Abstract] OR “pattern recognition systems”[Title/Abstract] OR (comput*[Title/Abstract] AND “pattern recognition”[Title/Abstract]) OR “k Nearest Neighbor”[Title/Abstract] OR “K nearest neighbour”[Title/Abstract] OR “random forest”[Title/Abstract] | 735,019 |
| #3 | #1 OR #2 | 801,103 |
| #4 | “GRADE approach”[MeSH Terms] | 130 |
| #5 | “GRADE approach”[Title/Abstract] OR “evidence grading system”[Title/Abstract] OR “GRADE assessment”[Title/Abstract] OR “evidence classification”[Title/Abstract] OR “evidence appraisal”[Title/Abstract] OR “evidence level”[Title/Abstract] OR “evidence grade”[Title/Abstract] OR “recommendation grade”[Title/Abstract] OR “GRADE method”[Title/Abstract] OR “GRADE framework”[Title/Abstract] OR “GRADE evidence”[Title/Abstract] OR “GRADE grading”[Title/Abstract] OR “quality of evidence”[Title/Abstract] OR “evidence strength”[Title/Abstract] OR “evidence-based assessment”[Title/Abstract] OR “quality assessment” [Title/Abstract] OR “risk of bias”[Title/Abstract] | 91,563 |
| #6 | #4 OR #5 | 91,581 |
| #7 | #3 AND #6 | 833 |
| **Cochrane Central Register of Controlled Trials** | |  |
| #1 | MeSH descriptor: [artificial intelligence] explode all trees | 3,348 |
| #2 | MeSH descriptor: [machine learning] explode all trees | 1,035 |
| #3 | MeSH descriptor: [neural networks] explode all trees | 552 |
| #4 | ((algorit*) or “artificial intelligence” or AI or “computational intelligence” or “machine intelligence” or “computer reasoning” or (computer vision*) or (machine learnin*) or (deep learnin*) or (neural network*) or “computer-assisted” or “computer-aided” or (“large language model”) or (support vector machine*) or (support vector network*) or “computer methodologies” or “automated pattern recognition” or “pattern recognition systems” or (comput* and “pattern recognition”) or “k Nearest Neighbor” or “K nearest neighbour” or “random forest”): ti,ab,kw | 35,611 |
| #5 | #1 OR #2 OR #3 OR #4 | 37,287 |
| #6 | MeSH descriptor: [GRADE approach] explode all trees | 3 |
| #7 | (“GRADE approach” or “evidence grading system” or “GRADE assessment” or “evidence classification” or “evidence appraisal” or “evidence level” or “evidence grade” or “recommendation grade” or “GRADE method” or “GRADE framework” or “GRADE evidence” or “GRADE grading” or “quality of evidence” or “quality assessment” or “evidence strength” or “evidence-based assessment” or “risk of bias”): ti,ab,kw | 9,389 |
| #8 | #6 OR #7 | 9,389 |
| #9 | #5 AND #8 | 671 |
| **EMbase** | |  |
| #1 | ‘artificial intelligence’(topic)’/exp OR ‘machine learning’ (topic)’/exp OR ‘neural networks’ (topic)’/exp | 571,728 |
| #2 | ‘algorit*’:ti,ab,kw OR ‘artificial intelligence’:ti,ab,kw OR AI:ti,ab,kw OR ‘computational intelligence’:ti,ab,kw OR ‘machine intelligence’:ti,ab,kw OR ‘computer reasoning’:ti,ab,kw OR ‘computer vision*’:ti,ab,kw OR ‘machine learnin*’:ti,ab,kw OR ‘deep learnin*’:ti,ab,kw OR ‘neural network*’:ti,ab,kw OR ‘computer-assisted’:ti,ab,kw OR ‘computer-aided’:ti,ab,kw OR ‘large language model’:ti,ab,kw OR ‘support vector machine*’:ti,ab,kw OR ‘support vector network*’:ti,ab,kw OR ‘computer methodologies’:ti,ab,kw OR ‘automated pattern recognition’:ti,ab,kw OR ‘pattern recognition systems’:ti,ab,kw OR (comput*:ti,ab,kw AND ‘pattern recognition’:ti,ab,kw) OR ‘k Nearest Neighbor’:ti,ab,kw OR ‘K nearest neighbour’:ti,ab,kw OR ‘random forest’:ti,ab,kw | 333,107 |
| #3 | #1 OR #2 | 631,307 |
| #4 | ‘GRADE approach’ (topic)'/exp | 4,966 |
| #5 | ‘GRADE approach’:ti,ab,kw OR ‘evidence grading system’:ti,ab,kw OR ‘GRADE assessment’:ti,ab,kw OR ‘evidence classification’:ti,ab,kw OR ‘evidence appraisal’:ti,ab,kw OR ‘evidence level’:ti,ab,kw OR ‘evidence grade’:ti,ab,kw OR ‘recommendation grade’:ti,ab,kw OR ‘GRADE method’:ti,ab,kw OR ‘GRADE framework’:ti,ab,kw OR ‘GRADE evidence’:ti,ab,kw OR ‘GRADE grading’:ti,ab,kw OR ‘quality of evidence’:ti,ab,kw OR ‘quality assessment’:ti,ab,kw OR ‘evidence strength’:ti,ab,kw OR ‘evidence-based assessment’:ti,ab,kw OR ‘risk of bias’:ti,ab,kw | 101,249 |
| #6 | #4 OR #5 | 103.032 |
| #7 | #3 AND #6 | 513 |
| **Web of Science** | |  |
| #1 | TS= (“artificial intelligence” OR “machine learning” OR “neural networks”) | 1,522,406 |
| #2 | TI= (“algorit*” OR “artificial intelligence” OR AI OR “computational intelligence” OR “machine intelligence” OR “computer reasoning” OR “computer vision*” OR “machine learnin*” OR “deep learnin*” OR “neural network*” OR “computer-assisted” OR “computer-aided” OR “large language model” OR “support vector machine*” OR “support vector network*” OR “computer methodologies” OR “automated pattern recognition” OR “pattern recognition systems” OR (comput* AND “pattern recognition” ) OR “k Nearest Neighbor” OR “K nearest neighbour” OR “random forest”) | 1,262,387 |
| #3 | #1 OR #2 | 2,215,646 |
| #4 | TS= (“GRADE approach”) | 5,173 |
| #5 | TI= (“GRADE approach” OR “evidence grading system” OR “GRADE assessment” OR “evidence classification” OR “evidence appraisal” OR “evidence level” OR “evidence grade” OR “recommendation grade” OR “GRADE method” OR “GRADE framework” OR “GRADE evidence” OR “GRADE grading” OR “quality of evidence” OR “quality assessment” OR “evidence strength” OR “evidence-based assessment” OR “risk of bias”) | 1,894 |
| #6 | #4 OR #5 | 6,885 |
| #7 | #3 AND #6 | 697 |
| **IEEE Xplore** | | |
| #1 | "Mesh_Terms":"artificial intelligence" OR "Mesh_Terms":"machine learning" OR "Mesh_Terms":"neural networks" | 16,374 |
| #2 | "Document Title":"algorit*" OR "Document Title":"artificial intelligence" OR "Document Title":AI OR "Document Title":"computational intelligence" OR "Document Title":"machine intelligence" OR "Document Title":"computer reasoning" OR "Document Title":"computer vision*" OR "Document Title":"machine learnin*" OR "Document Title":"deep learnin*" OR "Document Title":"neural network*" OR "Document Title":"computer-assisted" OR "Document Title":"computer-aided" OR "Document Title":"large language model" OR "Document Title":"support vector machine*" OR "Document Title":"support vector network*" OR "Document Title":"computer methodologies" OR "Document Title":"automated pattern recognition" OR "Document Title":"pattern recognition systems" OR "Document Title":"k Nearest Neighbor" OR "Document Title":"K nearest neighbour" OR "Document Title":"random forest" | 556,463 |
| #3 | "Document Title":comput* AND "Document Title":"pattern recognition" | 155 |
| #4 | #1 OR #2 OR #3 | 568,260 |
| #5 | "Mesh_Terms":"GRADE approach" OR "Document Title":"GRADE approach" OR "Document Title":"evidence grading system" OR "Document Title":"GRADE assessment" OR "Document Title":"evidence classification" OR "Document Title":"evidence appraisal" OR "Document Title":"evidence level" OR "Document Title":"evidence grade" OR "Document Title":"recommendation grade" OR "Document Title":"GRADE method" OR "Document Title":"GRADE framework" OR "Document Title":"GRADE evidence" OR "Document Title":"GRADE grading" OR "Document Title":"quality of evidence" OR "Document Title":"quality assessment" OR "Document Title":"evidence strength" OR "Document Title":"evidence-based assessment" OR "Document Title":"risk of bias" | 5,171 |
| #6 | #4 AND #5 | 627 |
| **ACM Digital Library** | | |
| #1 | [Title: "algorit*"] OR [Title: "artificial intelligence"] OR [Title: ai] OR [Title: "computational intelligence"] OR [Title: "machine intelligence"] OR [Title: "computer reasoning"] OR [Title: "computer vision*"] OR [Title: "machine learnin*"] OR [Title: "deep learnin*"] OR [Title: "neural network*"] OR [Title: "computer-assisted"] OR [Title: "computer-aided"] OR [Title: "large language model"] OR [Title: "support vector machine*"] OR [Title: "support vector network*"] OR [Title: "computer methodologies"] OR [Title: "automated pattern recognition"] OR [Title: "pattern recognition systems"] OR [Title: "k nearest neighbor"] OR [Title: "k nearest neighbour"] OR [Title: "random forest"] OR [Title: "computer pattern recognition"] | 77,526 |
| #2 | [Title: "grade approach"] OR [Title: "evidence grading system"] OR [Title: "grade assessment"] OR [Title: "evidence classification"] OR [Title: "evidence appraisal"] OR [Title: "evidence level"] OR [Title: "evidence grade"] OR [Title: "recommendation grade"] OR [Title: "grade method"] OR [Title: "grade framework"] OR [Title: "grade evidence"] OR [Title: "grade grading"] OR [Title: "quality of evidence"] OR [Title: "quality assessment"] OR [Title: "evidence strength"] OR [Title: "evidence-based assessment"] OR [Title: "risk of bias"] | 2,583 |
| #3 | #1 AND #2 | 104 |
| **中国知网（CNKI）** | | |
| #1 | SU= ((人工智能+机器学习+深度学习+神经网络+自然语言处理+大语言模型+计算机视觉+模式识别+智能算法+智能系统+监督学习+支持向量机+决策树+K近邻算法 +图像识别+数据挖掘)*( 证据分级+证据水平+质量评价+分级体系+证据评价+GRADE系统+分级系统+质量分级+循证分级)) | 346 |
| **维普(VIP)** | | |
| #1 | U=((人工智能OR 机器学习 OR 深度学习 OR 神经网络 OR 自然语言处理 OR 大语言模型 OR 计算机视觉 OR 模式识别 OR 智能算法 OR 智能系统 OR 监督学习 OR 支持向量机 OR 决策树 OR K近邻算法 OR k-NN OR 图像识别 OR 数据挖掘) AND (证据分级 OR 证据水平 OR 分级体系 OR 证据评价 OR 质量评价 OR GRADE系统 OR 分级系统 OR 质量分级 OR 循证分级)) | 211 |
| **万方(Wanfang Data)** | | |
| #1 | 主题: ((人工智能 or 机器学习 or 深度学习 or 神经网络 or 自然语言处理 or 大语言模型 or 计算机视觉 or 模式识别 or 智能算法 or 智能系统 or 监督学习 or 支持向量机 or 决策树 or K近邻算法 or 图像识别 or 数据挖掘) AND ( 证据分级 or 证据水平 or 分级体系 or 证据评价 or GRADE系统 or 分级系统 or 质量分级 or 质量评价 or 循证分级)) | 205 |
| **中国生物医学文献数据库(CBM)** | | |
| #1 | “人工智能”[不加权:扩展] OR “机器学习” [不加权:扩展] OR “神经网络”[不加权:扩展] | 4515 |
| #2 | “人工智能”[常用字段:智能] OR “机器学习” [常用字段:智能] OR “深度学习”[常用字段:智能] OR “神经网络”[常用字段:智能] OR “自然语言处理”[常用字段:智能] “大语言模型”[常用字段:智能] OR “计算机视觉”[常用字段:智能] OR “模式识别” [常用字段:智能] OR “智能算法”[常用字段:智能] OR “智能系统”[常用字段:智能] OR “监督学习”[常用字段:智能] OR “支持向量机”[常用字段:智能] OR “决策树”[常用字段:智能] OR “K近邻算法”[常用字段:智能] OR “图像识别”[常用字段:智能] OR “数据挖掘”[常用字段:智能] | 49437 |
| #3 | #1 OR #2 | 49437 |
| #4 | “证据分级”[不加权:扩展] OR “证据水平”[不加权:扩展] OR “分级体系”[不加权:扩展] OR “证据评价”[不加权:扩展] OR “GRADE系统”[不加权:扩展] OR “分级系统”[不加权:扩展] OR “质量分级”[不加权:扩展] OR “质量评价”[不加权:扩展] OR “循证分级”[不加权:扩展] | 860 |
| #5 | “证据分级”[常用字段:智能] OR “证据水平”[常用字段:智能] OR “分级体系”[常用字段:智能] OR “证据评价”[常用字段:智能] OR “GRADE系统”[常用字段:智能] OR “分级系统”[常用字段:智能] OR “质量分级”[常用字段:智能] OR “质量评价”[常用字段:智能] OR “循证分级”[常用字段:智能] | 1323 |
| #6 | #4 OR #5 | 1428 |
| #7 | #3 AND #6 | 186 |

Table S2 Basic characteristics of studies on automated support tools for evidence quality assessment

| **Study** | **Country** | **Study Type** | **Tool Name** | **Methodological Standard** | **Technology Implemented** | **Intended Task** | **Application Context** | **Tool Link** |
| --- | --- | --- | --- | --- | --- | --- | --- | --- |
| Armijo-Olivo 2020[1] | Canada | Observational Study | RobotReviewer (Public Access) | Cochrane RoB Tool | Machine Learning Algorithms (binary classification), NLP | Automatically assess risk of bias in RCTs to expedite SRs, HTAs, etc. | Performed risk of bias assessment on 372 physical therapy RCTs | [https://www.robotreviewer.net/](https://www.robotreviewer.net/?utm_source=chatgpt.com) |
| Hirt 2021[2] | Switzerland | Observational Study | RobotReviewer (Public Access) | Cochrane RoB Tool | Machine Learning Algorithms (binary classification), NLP | Automatically assess risk of bias in RCTs to expedite SRs, HTAs, etc. | Performed risk of bias assessment on 190 RCTs from nursing-related Cochrane reviews | [https://www.robotreviewer.net/](https://www.robotreviewer.net/?utm_source=chatgpt.com) |
| Edwards 2017[3] | United Kingdom | Observational Study | RobotReviewer (Public Access) | Cochrane RoB Tool | Machine Learning Algorithms (binary classification), NLP | Automatically assess risk of bias in RCTs to expedite SRs, HTAs, etc. | Performed risk of bias assessment on 35 RCTs | https://www.robotreviewer.net/ |
| Brassey 2021[4] | United Kingdom | Observational Study | Evidence Mapping Tool  (Public Access) | Cochrane RoB Tool | Machine Learning (binary classification), NLP and rule-based systems | Automatically identify, assess and collate intervention study evidence for evidence-based medicine | Performed risk of bias assessment on RCTs | https://evidencemap.com/ |
| Arno 2022[5] | United Kingdom | Randomized Controlled Trial | RobotReviewer (Public Access) | Cochrane RoB Tool | Machine Learning Algorithms (binary classification), NLP | Automatically assess risk of bias in RCTs to expedite SRs, HTAs, etc. | Assisted systematic reviewers in completing Cochrane risk of bias assessments | https://www.robotreviewer.net/ |
| Clark 2020[6] | Australia | Observational Study | RobotReviewer (Public Access) | Cochrane RoB Tool | Machine Learning Algorithms (binary classification), NLP | Automatically assess risk of bias in RCTs to expedite SRs, HTAs, etc. | Accelerated risk of bias assessment on 8 RCTs in a SR of increased fluid intake for urinary tract infection prevention | https://www.robotreviewer.net/ |
| Gates 2018[7] | Canada | Observational Study | RobotReviewer (Public Access) | Cochrane RoB Tool | Machine Learning Algorithms (binary classification), NLP | Automatically assess risk of bias in RCTs to expedite SRs, HTAs, etc. | Performed risk of bias assessment on 1180 RCTs across 10 clinical topics | https://www.robotreviewer.net/ |
| Gates 2021[8] | Canada | Observational Study | ExaCT | Cochrane RoB Tool | Text Mining, NLP | Automatically extract study characteristics from publications of randomized trials | Performed risk of bias assessment on RCTs | http://exactdemo.iit.nrc.ca |
| Park 2018[9] | United Kingdom | Methodological Study | RobotReviewer (Public Access) | Cochrane RoB Tool | Machine Learning Algorithms, NLP | Automatically assess risk of bias in RCTs to expedite SRs, HTAs, etc. | Performed risk of bias assessment on RCTs | https://www.robotreviewer.net/ |
| Zurrer 2024[10] | Switzerland | Observational Study | STEED | Cochrane RoB Tool | R Programming, Data Mining, Text Mining, Rule Matching | Automated extraction of experimental parameters and risk of bias items from neuroscience in vivo publications | Extracted experimental parameters and risk of bias items from animal studies of motor neuron diseases and multiple sclerosis. | / |
| Woelfle 2024[11] | Switzerland | Observational Study | Large Language Models | A MeaSurement Tool to Assess systematic Reviews (AMSTAR), Preferred Reporting Items for Systematic reviews and Meta-Analyses (PRISMA) | Deep Learning, Transformer Architecture | Advanced natural language processing and reasoning, with broad applicability across text-based evaluation and evidence appraisal | Assessed reporting quality , methodological rigor, and trial pragmatism of systematic reviews and randomized controlled trials | / |
| Suster 2023[12] | Australia | Observational Study | EvidenceGRADEr  (Public Access) | Grading of Recommendation, Assessment, Development, and Evaluation (GRADE) | Machine Learning Algorithms (binary classification, multiclass classification, multilabel classification), Deep Learning, NLP | Automated quality assessment of medical evidence in systematic reviews | Applied to predict GRADE quality grades and downgrading criteria | / |
| Sarker 2015[13] | Australia | Observational Study | Automatic Evidence Quality Prediction (Not Named) | Strength of Recommendation Taxonomy (SORT) | Supervised Learning, Rule Matching, NLP | Predict the quality of medical evidence using supervised machine learning on abstract texts and meta-data | Performed automatic quality grading for 1132 evidence-based recommendations | / |
| Brozek 2008[14] | Canada | Methodological Study | GRADEpro (Public Access) | Grading of Recommendation, Assessment, Development, and Evaluation (GRADE) | Web Technologies, API Integration, GRADE Framework | Assess evidence quality, formulate recommendation strength, and generate summary tables and decision frameworks | Not specifically applied in a single research study | https://www.gradepro.org/ |
| Marshall 2016[15] | United Kingdom | Observational Study | RobotReviewer (Public Access) | Cochrane RoB Tool | Machine Learning Algorithms (binary classification), NLP | Automatically assess risk of bias in RCTs to expedite SRs, HTAs, etc. | Performed risk of bias assessment on RCTs | https://www.robotreviewer.net/ |
| Kwong 2023[16] | Canada | Observational Study | APPRAISE-AI | Quality Assessment of Diagnostic Accuracy Studies (QUADAS-2), Transparent Reporting of a Multivariable Prediction Model for Individual Prognosis or Diagnosis (TRIPOD) | Quality Evaluation Framework, Statistical Analysis | Evaluate the methodological and reporting quality of AI prediction models for clinical decision support | Assessed overall quality of 28 machine learning studies for sepsis prediction | / |
| Jardim 2022[17] | Norway | Randomized Controlled Trial | RobotReviewer (Public Access) | Cochrane RoB Tool | Machine Learning Algorithms (binary classification), NLP | Risk of bias assessment for RCTs with extraction of supporting informative text | Performed risk of bias assessment on 26 RCTs in health and social interventions | https://www.robotreviewer.net/ |
| Sterne 2019[18] | United Kingdom | Methodological Study | ROB 2 Excel Macro Tool (Public Access) | Cochrane RoB 2 Tool | Excel VBA, Logic Rules, Form Controls | Ggenerate domain-level risk of bias judgments via signaling question algorithms, and produce summary tables and risk of bias graphs | Performed risk of bias assessment on RCTs | / |
| Eisele-Metzger 2025[19] | Germany | Observational Study | Claude 2 | Cochrane RoB 2 Tool | Large Language Models, Transformer Architecture | Assist complex text analysis through natural language processing, applicable to medical research requiring large-scale text comprehension | Performed risk of bias assessment on 100 RCTs focusing on the healthcare field | / |
| Kuitunen 2024[20] | Finland | Observational Study | ChatGPT | Cochrane RoB 2 Tool | Large Language Models, NLP | Achieve natural language understanding, generation, and dialogue interaction in various areas | Performed risk of bias assessment in evidence-based medicine | / |

“/” indicates that the tool is either not publicly available or still under development, and no available link exists.

**References for included studies**

1.	Armijo-Olivo S, Craig R, Campbell S: **Comparing machine and human reviewers to evaluate the risk of bias in randomized controlled trials**. *Res Synth Methods* 2020, **11**(3):484-493.

2.	Hirt J, Meichlinger J, Schumacher P, Mueller G: **Agreement in Risk of Bias Assessment Between RobotReviewer and Human Reviewers: An Evaluation Study on Randomised Controlled Trials in Nursing-Related Cochrane Reviews**. *J Nurs Scholarsh* 2021, **53**(2):246-254.

3.	Edwards M, Marshall C: **Evaluating robotreviewer for automated risk of bias assessment in a systematic review: A case study**. *Value in Health* 2017, **20**(9):A774-A774.

4.	Brassey J, Price C, Edwards J, Zlabinger M, Bampoulidis A, Hanbury A: **Developing a fully automated evidence synthesis tool for identifying, assessing and collating the evidence**. *BMJ Evid Based Med* 2021, **26**(1):24-27.

5.	Arno A, Thomas J, Wallace B, Marshall IJ, McKenzie JE, Elliott JH: **Accuracy and Efficiency of Machine Learning-Assisted Risk-of-Bias Assessments in "Real-World" Systematic Reviews : A Noninferiority Randomized Controlled Trial**. *Ann Intern Med* 2022, **175**(7):1001-1009.

6.	Clark J, Glasziou P, Del Mar C, Bannach-Brown A, Stehlik P, Scott AM: **A full systematic review was completed in 2 weeks using automation tools: a case study**. *J Clin Epidemiol* 2020, **121**:81-90.

7.	Gates A, Vandermeer B, Hartling L: **Technology-assisted risk of bias assessment in systematic reviews: a prospective cross-sectional evaluation of the RobotReviewer machine learning tool**. *J Clin Epidemiol* 2018, **96**:54-62.

8.	Gates A, Gates M, Sim S, Elliott SA, Pillay J, Hartling L: **Creating efficiencies in the extraction of data from randomized trials: a prospective evaluation of a machine learning and text mining tool**. *BMC Med Res Methodol* 2021, **21**(1):169.

9.	Park SE, Thomas J: **Evidence synthesis software**. *BMJ Evid Based Med* 2018, **23**(4):140-141.

10.	Zurrer WE, Cannon AE, Ewing E, Brüschweiler D, Bugajska J, Hild BF, Rosso M, Reich DS, Ineichen BV: **STEED: A data mining tool for automated extraction of experimental parameters and risk of bias items from in vivo publications**. *PLoS One* 2024, **19**(11):e0311358.

11.	Woelfle T, Hirt J, Janiaud P, Kappos L, Ioannidis JPA, Hemkens LG: **Benchmarking Human-AI collaboration for common evidence appraisal tools**. *J Clin Epidemiol* 2024, **175**:111533.

12.	Šuster S, Baldwin T, Lau JH, Jimeno Yepes A, Martinez Iraola D, Otmakhova Y, Verspoor K: **Automating Quality Assessment of Medical Evidence in Systematic Reviews: Model Development and Validation Study**. *J Med Internet Res* 2023, **25**:e35568.

13.	Sarker A, Mollá D, Paris C: **Automatic evidence quality prediction to support evidence-based decision making**. *Artif Intell Med* 2015, **64**(2):89-103.

14.	Brozek J, Oxman A, Schünemann H: **GRADEpro.[Computer program]. Version 3.2 for Windows**. In*.*; 2008.

15.	Marshall IJ, Kuiper J, Wallace BC: **RobotReviewer: evaluation of a system for automatically assessing bias in clinical trials**. *J Am Med Inform Assoc* 2016, **23**(1):193-201.

16.	Kwong JCC, Khondker A, Lajkosz K, McDermott MBA, Frigola XB, McCradden MD, Mamdani M, Kulkarni GS, Johnson AEW: **APPRAISE-AI Tool for Quantitative Evaluation of AI Studies for Clinical Decision Support**. *JAMA Netw Open* 2023, **6**(9):e2335377.

17.	Jardim PSJ, Rose CJ, Ames HM, Echavez JFM, Van de Velde S, Muller AE: **Automating risk of bias assessment in systematic reviews: a real-time mixed methods comparison of human researchers to a machine learning system**. *BMC Med Res Methodol* 2022, **22**(1):167.

18.	Sterne JAC, Savović J, Page MJ, Elbers RG, Blencowe NS, Boutron I, Cates CJ, Cheng HY, Corbett MS, Eldridge SM *et al*: **RoB 2: a revised tool for assessing risk of bias in randomised trials**. *BMJ* 2019, **366**:l4898.

19.	Eisele-Metzger A, Lieberum JL, Toews M, Siemens W, Heilmeyer F, Haverkamp C, Boehringer D, Meerpohl JJ: **Exploring the potential of Claude 2 for risk of bias assessment: Using a large language model to assess randomized controlled trials with RoB 2**. *Res Synth Methods* 2025, **16**(3):491-508.

20.	Kuitunen I, Ponkilainen VT, Liukkonen R, Nyrhi L, Pakarinen O, Vaajala M, Uimonen MM: **Evaluating the Performance of ChatGPT-4o in Risk of Bias Assessments**. *J Evid Based Med* 2024, **17**(4):700-702.
